# Supplementary material for: Effects of Chronic LY341495 on Hippocampal mTORC1 Signaling in Mice with Chronic Unpredictable Stress-Induced Depression
Source: Int J Mol Sci. 2022 Jun 8;23(12):6416. doi: 10.3390/ijms23126416 (PMC9224204; doi:10.3390/ijms23126416)
Supplement: Supplementary file 1 [file ijms-23-06416-s001.zip › ijms-1750567-supplementary.pdf]

Supplementary Information

List

Crude gels in Figure 2 --- 3

Crude gels in Figure 3 --- 8

Figure S1. Crude gels in Figure 2.

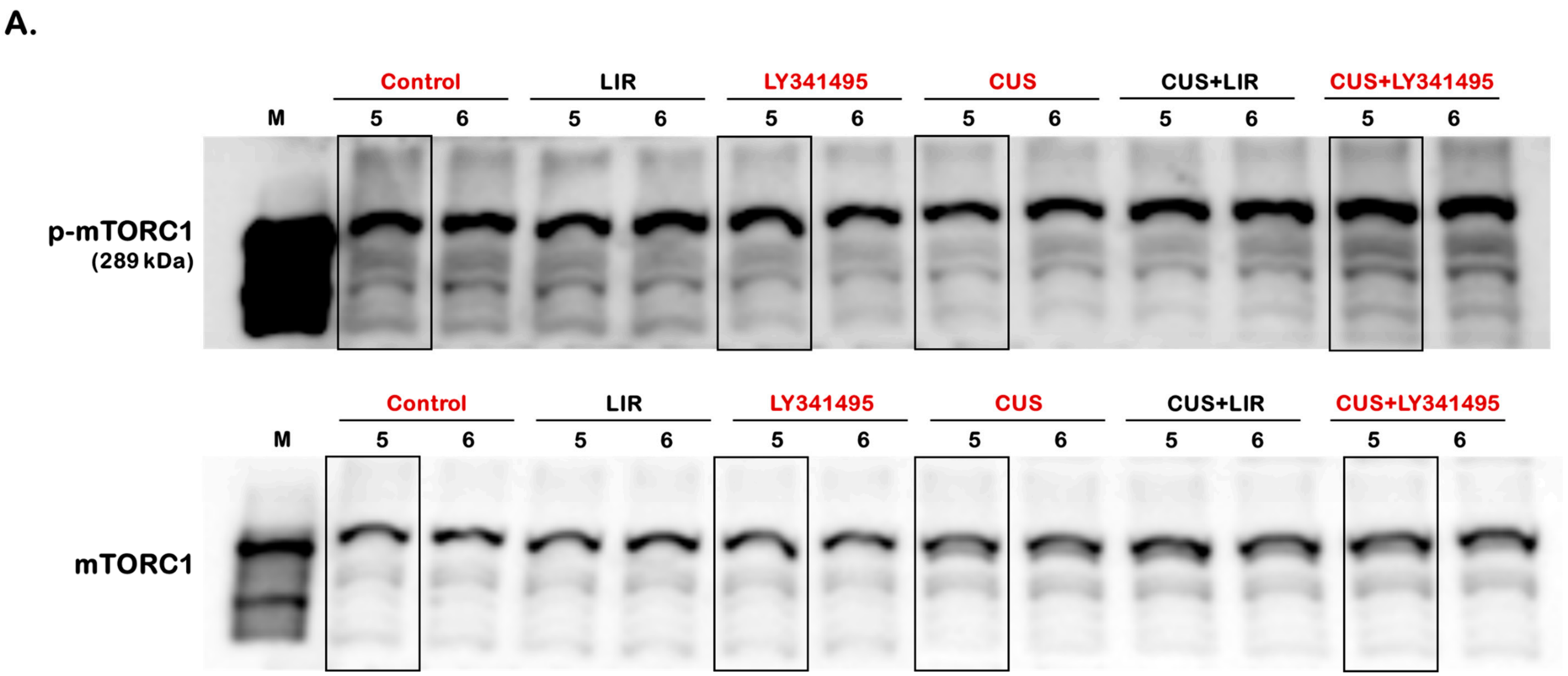

B.

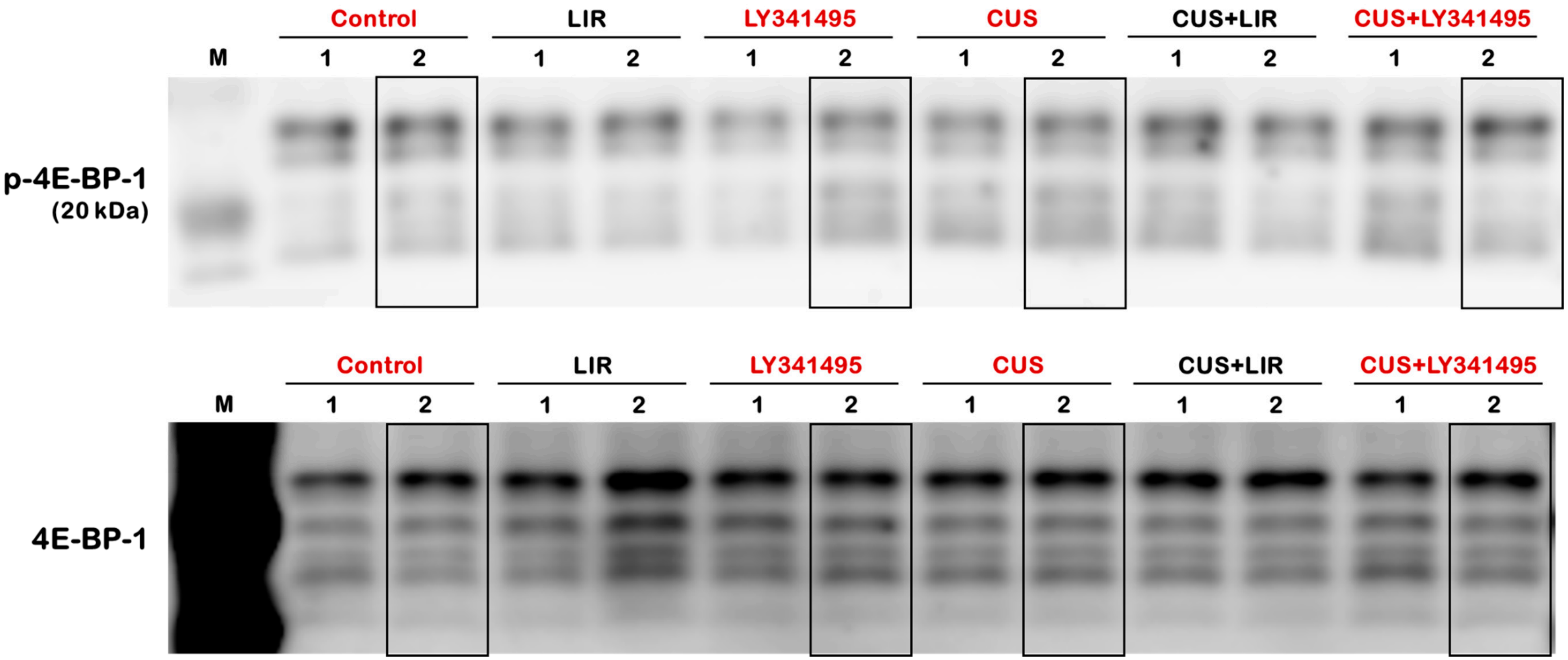
Control
LIR
LY341495
CUS
CUS+LIR
CUS+LY341495
M
1
2
p-4E-BP-1
(20 kDa)
4E-BP-1

C.

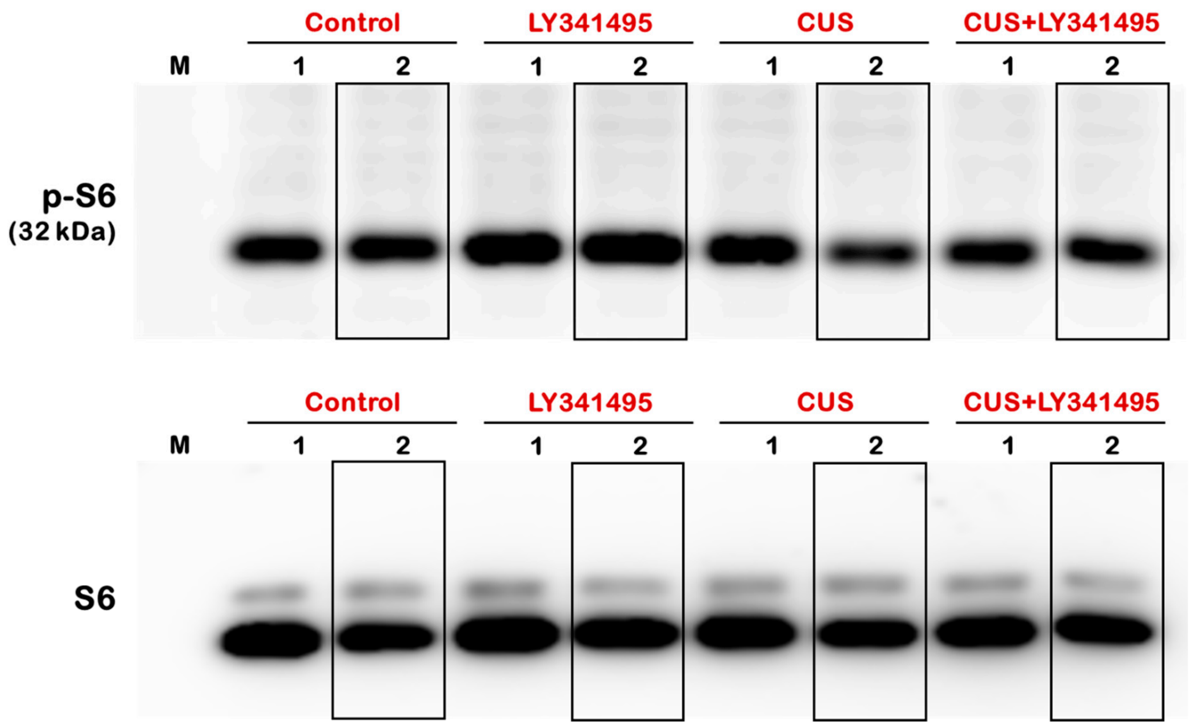

The original images from which we constructed Figures 2 A–C are shown below. Some wells are excluded because the relevant experimental groups (LIR and CUS+LIR) are not discussed in this paper.

A.

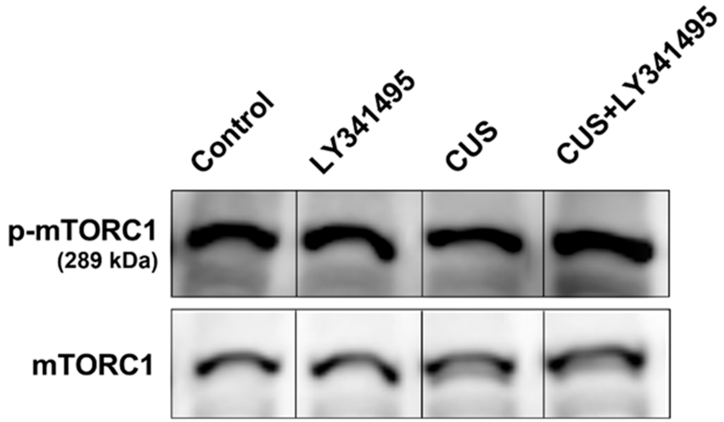
Control
LY341495
CUS
CUS+LY341495
p-mTORC1
(289 kDa)
mTORC1

B.

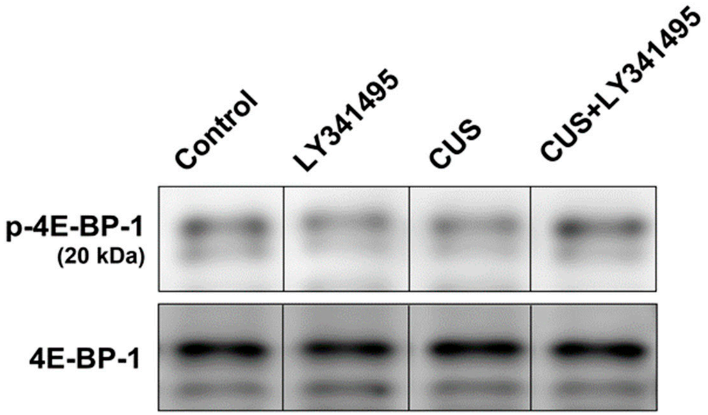
Control
LY341495
CUS
CUS+LY341495
p-4E-BP-1
(20 kDa)
4E-BP-1

C.

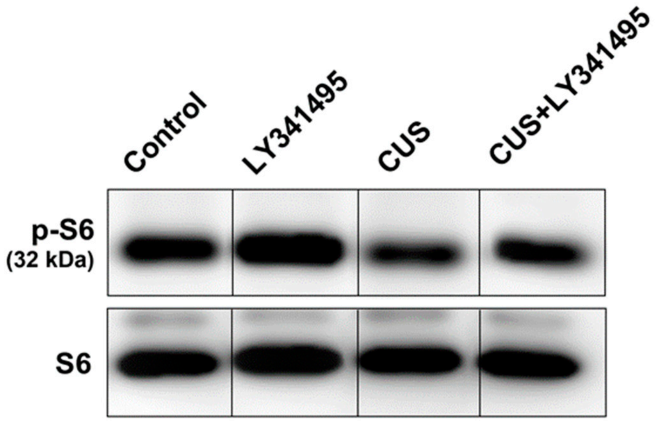

Figure S2. Effects of chronic LY341495 administration on the hippocampal levels of phosphorylated mTORC1, 4E-BP-1, and S6 in CUS-exposed mice.

Figure S2. Crude gels from Figure S3.

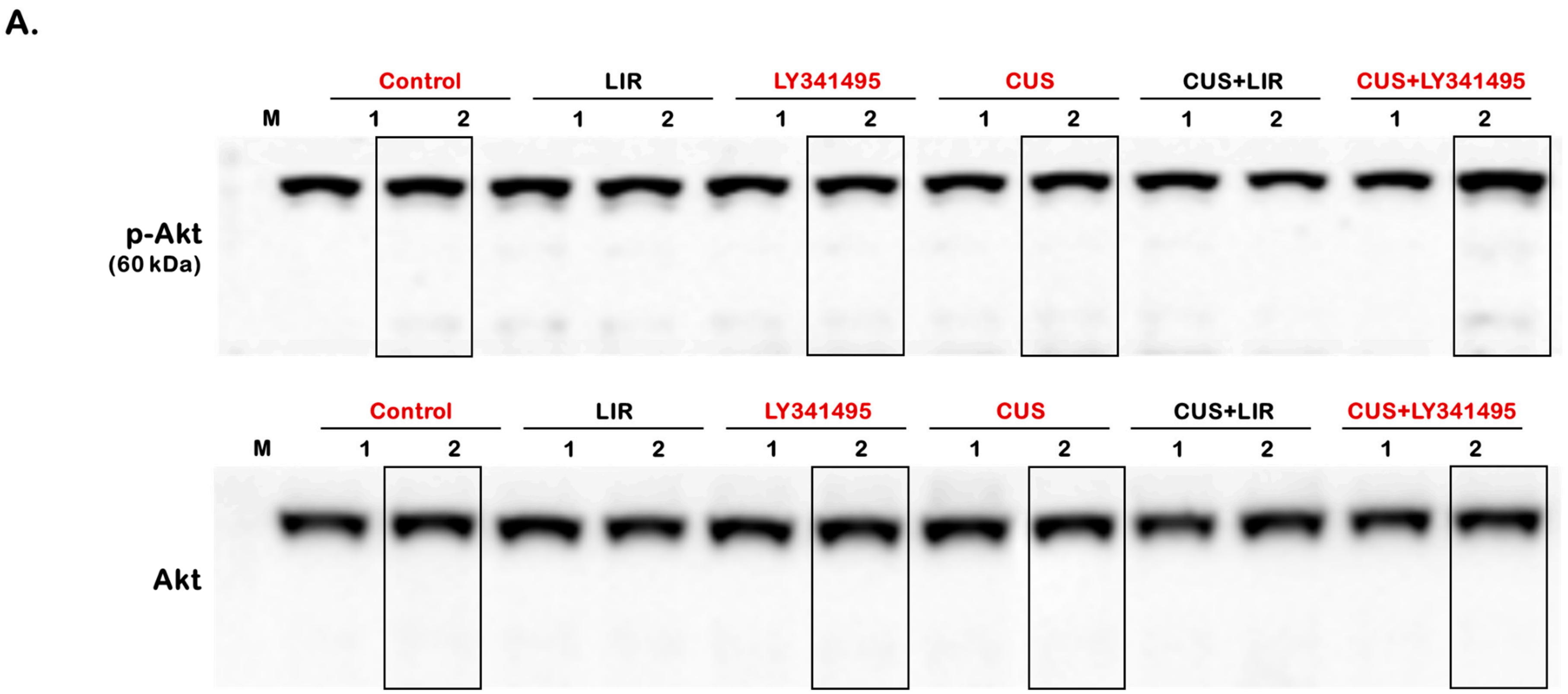

B.

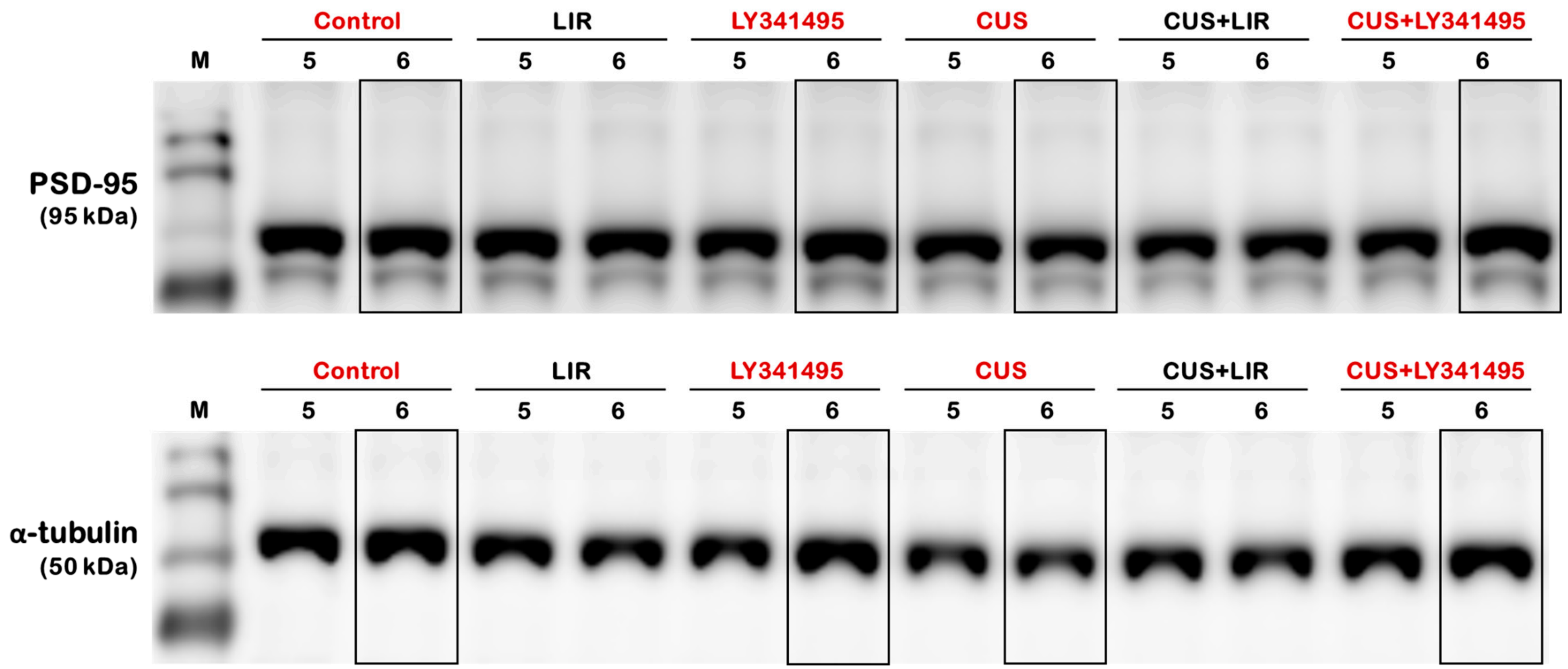
Control
LIR
LY341495
CUS
CUS+LIR
CUS+LY341495
M
5
6
PSD-95
(95 kDa)
Control
LIR
LY341495
CUS
CUS+LIR
CUS+LY341495
M
5
6
α-tubulin
(50 kDa)

C.

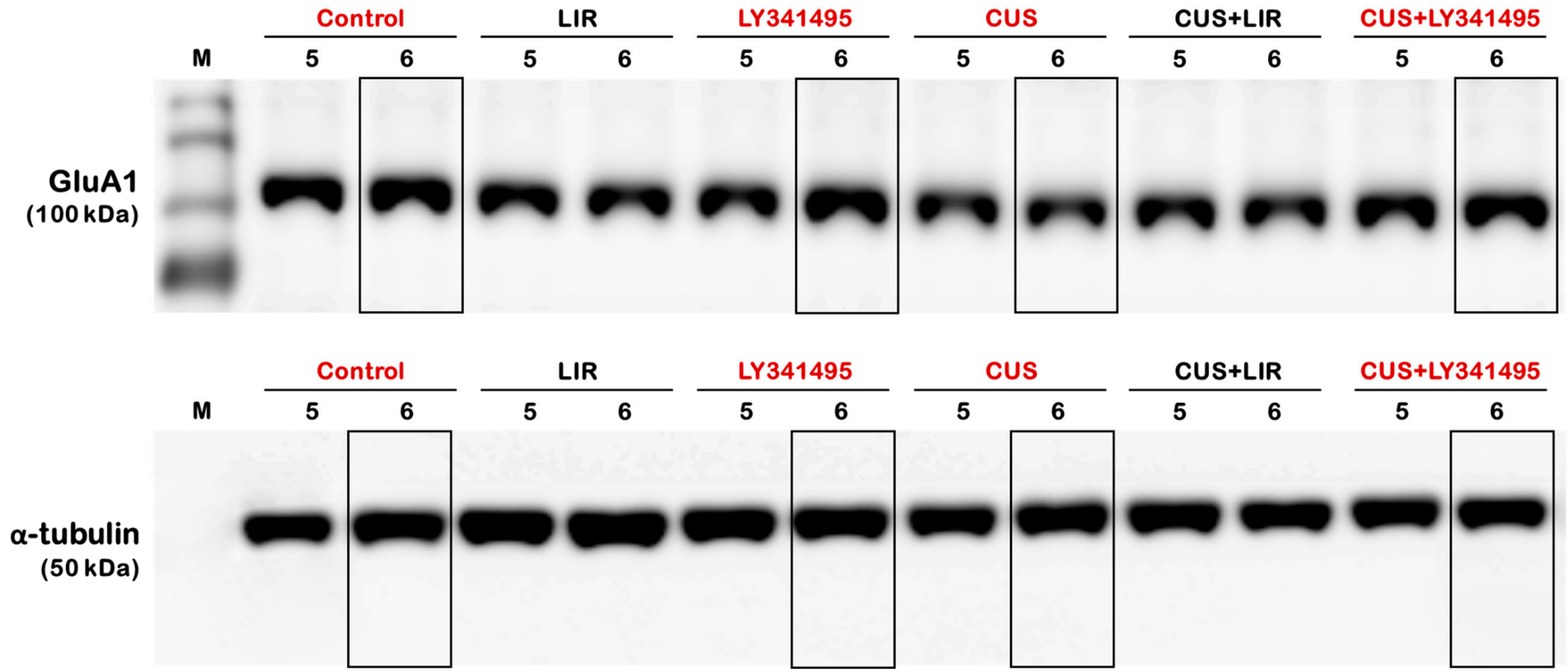

The original images from which we constructed Figures 3 A–C are shown below. Some wells are excluded because the relevant experimental groups (LIR and CUS+LIR) are not discussed in this paper.

A.

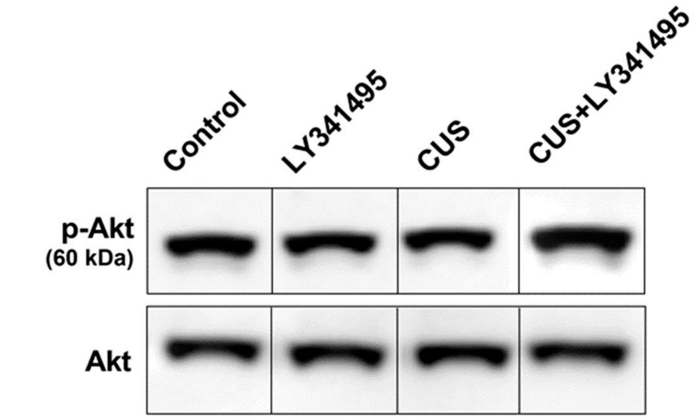
Control
LY341495
CUS
CUS+LY341495
p-Akt
(60 kDa)
Akt

B.

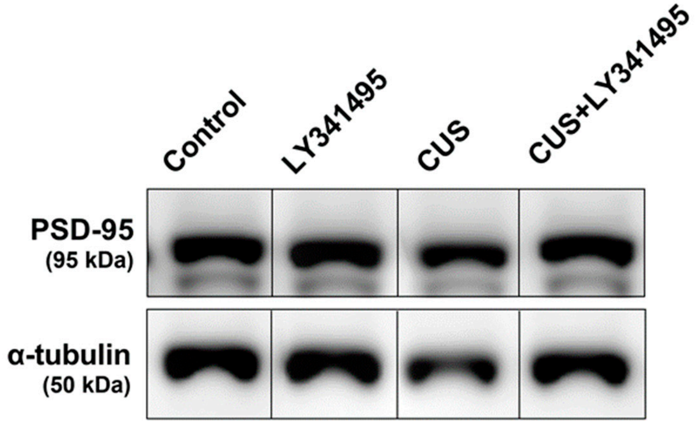
Control
LY341495
CUS
CUS+LY341495
PSD-95
(95 kDa)
α-tubulin
(50 kDa)

C.

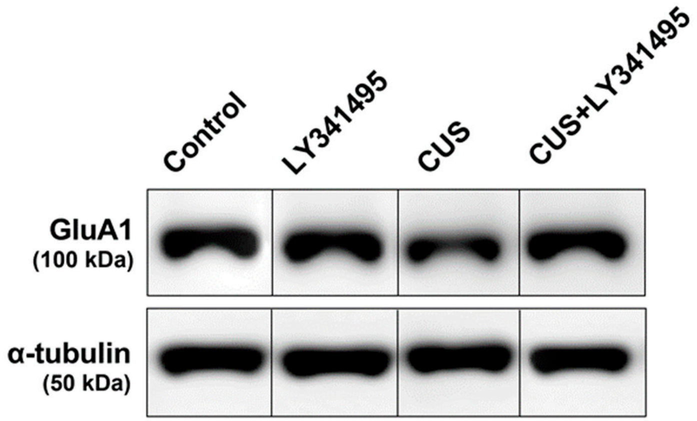

Figure S3. Effects of chronic LY341495 administration on Akt phosphorylation and PSD-95 and GluA1 hippocampal expression in CUS-exposed mice.
